# Wellness Within REACH

**Published:** 2006-06-15

**Authors:** African American Health Coalition

## About the Video

The African American Health Coalition, Inc was developed to implement initiatives that would reduce health disparities among African Americans and promote increased communication and trust between the African American community and local institutions and organizations. One of these initiatives is an annual Wellness Week featuring an African American Wellness Village and the Wellness within REACH Walk. The Wellness Village provides access to free health screenings, links between health care organizations and African American community members, and health education and information using a model of cultural sensitivity.

**Figure:**
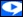
Watch* Wellness within REACH* (RM 15Mb) You will need RealOne Player to view these videos. Learn more about RealPlayer. Learn more about RealPlayer.

## Transcript

**Narrator**

For more than a decade, the African American Health Coalition watched as its volunteer efforts turned into a movement to improve health and save lives among African Americans in Oregon. The once-volunteer organization based in Portland is now a professional, community-based, multimillion-dollar nonprofit organization moving full force to make Portland's African American community the healthiest in the nation.

**Corliss McKeever, President, AAHC**

What we're really doing most effectively, I think, is listening to the community and letting them tell us what they're willing to do. You know, you can have the best program, the best designed program in the world, but if the community doesn't buy into it, or if they're not connected to it, it won't be effective.

**Narrator**

The work of improving health among African Americans is a challenge when you consider the statistics. In Oregon, African Americans die more often of preventable diseases than white Americans. It happens for a variety of reasons.

**Corliss McKeever**

Our vision is to be the healthiest African American community in the nation. When you think about the fact that we're less than 2% of the population in Oregon, and over 80% of that 2% is located in the Portland metro area, we can do this. We can get the word out.

**Narrator**

The number one killer of African Americans in Oregon is cardiovascular disease. To drive down those deadly statistics, the first annual Wellness Within Reach Walk was born in 2003. Hundreds took to the streets to promote better health among African Americans, and many pledged to improve their lifestyles long-term.

**Participant**

You know, I'm so happy I'm able to do it and I do have the health to do it. And I'm going to support this in every way I can.

**Narrator**

It's the Wellness Within Reach Mind, Body, and Soul Program, and it's thanks to a federal grant called REACH, or Racial and Ethnic Approaches to Community Health.

**Keith Dempsey, Program Manager [video says "Pgm Mgr"], Wellness Within Reach**

I guess the most exciting aspect is that people are taking a hold of this thing. And it's not just a program. We thought it was just a program. What it's starting to become is a movement.

**Aaron Hamlin, Senior Pastor**

It started out small, but it grew rapidly within a week. And it's really folks coming in from everywhere. In fact, there was no place to park.

**Narrator**

For Audrey Holt, a mother of five, this class has boosted her confidence and made her into a community example of healthy living.

**Audrey Holt, Mother of five**

And not just for me but for the elder women and men. We had a gentleman who was 80 years old in the class. And I just applauded him. I said, "if he can do it, my God, I know, I know." You know, he gave me inspiration. So I applaud, I applaud the program. I applaud it.

**Keith Dempsey**

From 21 years old to 87, folks are just saying, "I feel better about myself, I look better, and I feel like I can go on. I have hope." And that all stems from feeling better and doing better physically.

**Georgann Pierce, Instructor**

I'm seeing a consistency. I'm seeing more people are getting results, like one lady in the class in particular, she lost already 20 pounds, and that's just only through exercise. And so, I'm just saying it's paying off, it's paying off for a lot of people.

**Narrator**

Exercise is only one program aimed at improving health among African Americans. There are many more. The coalition tackles diabetes with its Linkage program that teaches better health through nutrition. More women are going for early detection breast cancer screening, thanks to our breast and cervical cancer program.

**Corliss McKeever**

Any time you have one race continuing to have poorer health outcomes than the dominant population, it is everybody's responsibility to be concerned, because it drives health care costs. And prevention is the key, and it's a way for everyone to benefit. It's a win-win situation for us to help prevent chronic illness and disease in African Americans, and we need everyone's help.

## Author Information

For more information about this video, Corliss McKeever, MSW, African American Health Coalition, Inc. Telephone: 503-413-1850. Email: corlissm@aahc-portland.org.

